# Immunosensor Based on Zinc Oxide Nanocrystals Decorated with Copper for the Electrochemical Detection of Human Salivary Alpha-Amylase

**DOI:** 10.3390/mi12060657

**Published:** 2021-06-03

**Authors:** Beatriz Rodrigues Martins, Tainá Marques Sampaio, Ana Karoline Silva Rocha de Farias, Rheltheer de Paula Martins, Renata Roland Teixeira, Robson Tadeu Soares Oliveira, Carlo Jose Freire Oliveira, Marcos Vinícius da Silva, Virmondes Rodrigues, Noelio Oliveira Dantas, Foued Salmen Espindola, Anielle Christine Almeida Silva, Renata Pereira Alves-Balvedi

**Affiliations:** 1 Physiological Science, Federal University of Triangulo Mineiro, Uberaba, MG 38025-180, Brazil; d201811482@uftm.edu.br (B.R.M.); rheltheer.martins@uftm.edu.br (R.d.P.M.); robson.junior@uftm.edu.br (R.T.S.O.J.); carlo.oliveira@uftm.edu.br (C.J.F.O.); marcos.silva@uftm.edu.br (M.V.d.S.); virmondes.rodrigues@uftm.edu.br (V.R.J.); 2 Biological Science, Federal University of Triangulo Mineiro, Iturama, MG 38280-180, Brazil; d201511169@uftm.edu.br (T.M.S.); d201710610@uftm.edu.br (A.K.S.R.d.F.); 3 Institute of Biotechnology, Federal University of Uberlandia, Uberlandia, MG 38405-319, Brazil; rolandteixeira@ufu.br (R.R.T.); foued@ufu.br (F.S.E.); 4 Laboratory of New Nanostructured and Functional Materials, Institute of Physics, Federal University of Alagoas, Maceió, AL 57072-900, Brazil; noelio@fis.ufal.br (N.O.D.); acalmeida@fis.ufal.br (A.C.A.S.); 5 Rede Nordeste de Biotecnologia (RENORBIO), Federal University of Alagoas, Maceió, AL 57072-900, Brazil

**Keywords:** zinc oxide nanoparticles, copper, nanocomposite, electrochemical detection, human salivary alpha-amylase, antibody, biomarker

## Abstract

(1) Background: Nanocrystals (NCs)-based electrochemical sensors have been proposed for biomarkers detection, although immunosensors using ZnO NCs decorated with copper are still scarce. (2) Methods: Electrochemical immunodetection of human salivary alpha-amylase (HSA) used ZnO, CuO, and ZnO:xCu (x = 0.1, 0.4, 1.0, 4.0, and 12.0) NCs. (3) Results: Substitutional incorporation of Cu^2+^ in the crystalline structure of ZnO and formation of nanocomposite were demonstrated by characterization. Graphite electrodes were used and the electrochemical signal increased by 40% when using ZnO:1Cu and 4Cu (0.25 mg·mL^−1^), in an immunosensor (0.372 mg·mL^−1^ of anti-alpha-amylase and 1% of casein). Different interactions of HSA with the alpha-amylase antibody were registered when adding the NCs together, either before or after the addition of saliva (4 μL). The immunosensor changed specificity due to the interaction of copper. The ZnO:1Cu and ZnO:4Cu samples showed 50% interference in detection when used before the addition of saliva. The immunosensor showed 100% specificity and a sensitivity of 0.00196 U·mL^−1^. (4) Conclusions: Results showed that the order of NCs addition in the sensors should be tested and evaluated to avoid misinterpretation in detection and to enable advances in the validation of the immunosensor.

## 1. Introduction

Nanoparticle research has been underway since the 1960s, with its first biological application in the 1980s [1]. There are odd properties associated with design, and this field presents a new universe with vast possibilities and enormous technological potential, mainly in the development of biosensor speed, low cost, and portability. Both organic (viral or protein) and inorganic (metal) nanoparticles exist in nature. However, they do not present controlled synthesis, which means a sample will not be homogeneous when collected at different sites or conditions [2,3].

Some reviews that have discussed scientific motivation to develop nanoparticles have applied progressively controllable synthesis techniques, used more sensitive characterization tools, and, finally, suggested theoretical models from experimental observations [4,5,6,7].

Among all different types of nanoparticles, zinc oxide (ZnO) has drawn more attention due to its remarkable properties and chemical features. Besides being an inexpensive material, it is already on the market as an agent for bioimaging and electrical devices with piezo and pyroelectrical properties [8,9,10]. Beyond the miniature size and the increase in surface area, bimetallic nanoparticles are better in catalyst form; they have attracted more attention than monometallic nanoparticles [11,12,13]. ZnO nanoparticles doped with other metals (Cu, Cr, N, Au, Ag, Al, Sn, Ga, Fe, Mn, and Co) become new materials with desired properties that cannot be achieved by monometallic nanoparticles [14,15,16]. In four decades of research, it has been found that the synthesis of these doped nanoparticles and nanocomposites can be evaluated from the electrochemical point of view, aiming at several technological applications [16,17,18].

The detection of HSA has been investigated as a stress biomarker in saliva, a fluid that is easy and non-invasive to collect. Studies of these biomarkers for both psychological stress and physical stress have been performed with the measurement of the enzymatic activity of alpha-amylase in the salivary fluid and its immunodetection by Western blot [19,20,21,22]. Electrochemical immunosensors, among others, have also been used for this purpose [23,24,25,26]. The incorporation of nanomaterials in biosensors aims to optimize detection performance, offering biocompatibility, additional connections, and electrical properties that improve signal strength.

An immunosensor to detect HSA using ZnO nanocrystals (NCs) decorated with Copper (Cu^2+^ ions) may be an attractive option due to the property of copper in interacting with salivary proteins, including HSA [27,28]. HSA is a calcium-binding protein, capable of binding two Ca^2+^ ions. One of the binding sites is exclusive to Ca^2+^; in the other enzyme site, the Cu^2+^ ions are bound by electrostatic interaction, like Ca^2+^ [28,29,30]. The interaction of CuO nanoparticles with the protease–amylase complex also revealed that the oxygen present in the nanoparticle forms hydrogen bonds and binds copper through van der Waals interactions to specific amino acids at the interaction site [31].

However, there are still several challenges for the development of sensors using electrochemical and optical methods with intelligent interfaces to evaluate biomarkers in salivary fluid, including those using antibodies and nanomaterials, such as the one in the present study [26]. Among the challenges are glassy carbon electrodes, as the working electrode constitutes the conventional electrochemical biosensor detection system [32] due to their larger size and the need for a surface treatment-limiting integration miniaturized portable design [33,34,35]. For the advancement of portable biosensor technologies capable of meeting the appeal of the advantages of using salivary fluid, such as the measurement of biomarkers in situ, some limitations can be overcome, such as using bench equipment and trained personnel for these analyzes [26].

Salivary fluid has been described as a complementary and potential fluid for the diagnosis of diseases and oxidative stress. Many pathologies and physiological changes caused, for example, by physical exercise have alterations in the oxidative stress biomarkers [36,37]. However, there are certain challenges, such as the need for sensitivities and resolution of the biosensors, since many of these biomarkers are in saliva in a much lower concentration than in plasma [38]. In addition, factors inherent to the salivary fluid, such as its secretion rate, pH, viscosity, and the complex environment of the oral cavity, can influence the determination of biomarkers [39,40,41,42].

In search of high sensitivity, response time, good performance, easy handling, accurate reporting, portability, low cost, and reliable detection of biomarkers, in this work, we developed an immunosensor based on ZnO NCs decorated with Cu for the electrochemical detection of HSA. Completing the research, we intended to evaluate the best interaction of these ZnO:xCu NCs with the sensor and the HSA available in the electrochemical sensor, confirming the entire process.

## 2. Materials and Methods

### 2.1. Reagents and Samples

Ultra-high purity water (deionized water, Milli-Q^®^ IX Water, Merck, Brazil) was used for the preparation of aqueous solutions, and all experiments were conducted at room temperature (25 ± 1 °C).

Reference [27] showed, for the first time, a bioelectrode based on the immobilization of a specific antibody for salivary alpha-amylase (Ab-0.744; 0.372 mg·mL^−1^). The blocking of the binding of non-specific biomolecules on the electrode surface was done with casein (1% and 10%), and it was stored at a temperature of −20 °C until use.

Seven types of NCs were used: 6 types of ZnO (pure, and with Cu (0.1, 0.4, 1, 4, 12)) and 1 type of CuO that was synthesized based on previous methodologies [17,18]. Stock solutions were prepared (0.5 mg·mL^−1^). The redox solution was 5 mM [Fe(CN)_6_]^−3/−4^ and 0.1 M KCl (FF/KCl), pH 7.4. All were stored at 4 °C until use.

Saliva samples from healthy human volunteers (n = 4, non-smokers) were collected in the morning, without stimulation, centrifuged (1500 rpm, 3 min), and stored at −20 °C until use for detection of human saliva alpha-amylase (HSA-Ag).

The alpha-amylase enzyme (AAE-Browin, Lódz, Polônia) and trypsin DPCC treated, type XI (TXI-Sigma-Aldrich, São Paulo, Brazil), were used for the specificity test and prepared in 0.25 mg·mL^−1^.

All samples were analyzed simultaneously in triplicate to assess repeatability and used as proof of concept.

Investigations were carried out following the rules of the Declaration of Helsinki of 1975, revised in 2013. According to point 23 of this declaration, approval from an ethics committee was obtained before undertaking the research. This study was conducted according to the ethical guidelines of the Brazilian Ministry of Health, with protocols and procedures approved by the Research Ethics Committee/UFTM, under protocol number 3.938.388, approved in 2020.

### 2.2. Apparatus for Characterization and Electrochemical Analysis

X-ray diffractograms (XRDs) were taken with an XRD-6000 (Shimadzu Corp., Tokyo, Japan), using monochromatic Cu-Kα1 (λ = 1.54056Å) radiation. The XDRs patterns confirmed ZnO and CuO NCs formation, crystal structure, average size, doping effects, and nanocomposite formation.

Analysis of the surface morphological characterization for a graphite electrode in the absence or presence of NCs was assessed through atomic force microscopy (AFM) Spm9600 (Shimadzu Corp., Tokyo, Japan).

To assess the conductivity of electrodes and for optimization of detection, cyclic voltammetry (CV) was used. The voltammetry measurements were performed using a Potentiostat EmStat1 (PalmSens, The Netherlands) with PSTrace 5.4 software (PalmSens, Houten, The Netherlands).

The measurements for detections were carried out in FF/KCl using a 100.0 mV·s^−1^, −0.8 and +0.8 V, 1scan, one-compartment cell, carbon screen-printed electrode (C110 220, DropSens, Spain). The working (WE) and auxiliary (AE) electrodes were made of carbon, while the reference electrode (RE) was made of silver. All electrochemical assays were conducted in triplicate.

### 2.3. Electrochemical Selection and Use of the Nanocrystals

Among types of ZnO NCs, we tested different dilutions to select the one with the best electrochemical conductivity onto the electrode. We used 4 μL of the NC solution on the surface of the WE for 20 min. Then, the electrode was washed with 100 μL of deionized water and dried in a vacuum dryer. Subsequently, 80 μL of FF/KCl was used to close the circuit (WE, RE, AE) and evaluate the electrochemical sign, by cyclic voltammetry (CV), for each type of NCs.

After that, we selected the NCs that best responded electrochemically and used them in the molecular recognition between the immobilized Ab and the monitored HSA-like antigen (Ag).

### 2.4. Immunosensor

The adsorption of anti-alpha-amylase polyclonal (Ab) as a probe was carried out by applying 4 μL on the WE surface. In the next step, the electrode received 4 μL of casein. After that, 4 μL of saliva (target-HSA) was applied to this same electrode with the antibody.

The procedure was performed to check the differences with the introduction of 4 μL NCs that best responded electrochemically: before, together, and after the application of human saliva.

After the sensor was set up, we evaluated its electrochemical signal, employing CV in one-compartment electrochemical cells connected to a potentiostat. These signals were measured by using 80 μL FF/KCl as a supporting electrolyte.

All the interactions were carried out at 25 ± 1 °C for 20 min, and at each step, the electrode was washed with deionized water and dried with a vacuum dryer.

## 3. Results

### 3.1. Characterization of ZnO:Cu Nanocrystals

**Figure 1:**
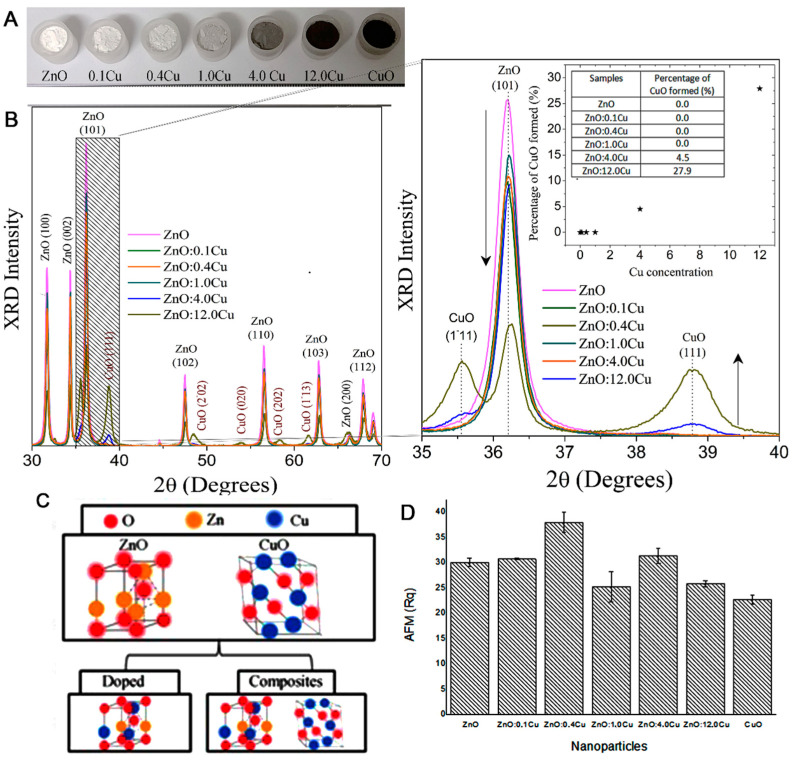
(**A**) Powdered samples, (**B**) XRD patterns of the ZnO samples with increasing Cu concentrations, (**C**) crystal structure of ZnO and CuO, exemplifying the doping process and composites, and (**D**) surface roughness analysis by AFM of the samples onto electrodes.

Figure 1 illustrates the following: (A) the change of color in powdered samples with doping, according to Cu^2+^ or CuO concentration; (B) the XRD patterns of the ZnO NC samples and of those samples with increasing concentrations of Cu; and (C) the crystal structure of the ZnO and CuO, subsequently exemplifying the doping process and composites in NCs. In Figure 1B, observed in the XRD results of the ZnO sample, narrow peaks confirmed the high crystallinity of the sample, and the Bragg diffraction peaks were characteristic of hexagonal wurtzite ZnO (JCPDS-EF 36-1451). The observed peaks for high concentrations of Cu correspond to single-phase CuO with a monoclinic structure (JCPDS-05-0661). The crystalline planes of ZnO and CuO were added. The zoom of the shaded region illustrates the changes with Cu concentration. For concentrations below ZnO:1Cu, only a major ZnO peak shift could be seen, indicating the substitutional incorporation of Cu^2+^ ions by Zn^2+^ ions into the ZnO crystal structure. Moreover, no additional peaks corresponding to CuO formation were observed, indicating that there was no significant CuO formation to be detected in the diffractograms. The concentrations above 1Cu exhibited the formation of a nanocomposite consisting of Cu-doped ZnO NCs and CuO NCs. The grain size was obtained from the XRD line-broadening measurement, using the Scherrer equation; being 20 nm, it confirmed the formation of NCs.

Figure 1C shows the crystal structure of ZnO and CuO, subsequently exemplifying the doping process and composites. The doping process consisted of the substitution of ions in the crystalline structure of the nanocrystal. The composite is a fascinating due synergism of properties [15]. Based on the XRD results, samples with concentrations of 0.1, 0.4, and 1.0 were Cu-doped ZnO NCs, and the concentrations of 4.0 and 12.0 were composites, i.e., the simultaneous presence of Cu-doped ZnO NCs and CuO NCs. Figure 1D shows the AFM results, whose surface roughness analysis (Rq) was used in the construction of the column chart. After adding different concentrations of NCs onto the electrode surface, the results showed that ZnO:0.4Cu (3th bar) presented 26.21% higher roughness compared with ZnO.

### 3.2. Electrochemical Analysis of ZnO:Cu Nanocrystals

The NCs’ electrochemical reactivity was evaluated with a redox probe FF/KCl through a donation of hydrogen and electrons during the anodic current (oxidation) and return hydrogen and electrons during the cathodic current (reduction).

Different NCs and concentrations (0.5, 0.25, 0.125, and 0.0625 mg·mL^−1^) were tested. Both currents may have increased or decreased when NCs were included on the electrode surface. To select the NCs and the optimal concentration, seven types of NC and different dilutions (0.5, 0.25, 0.125, and 0.062 mg·mL^−1^) were tested.

**Figure 2:**
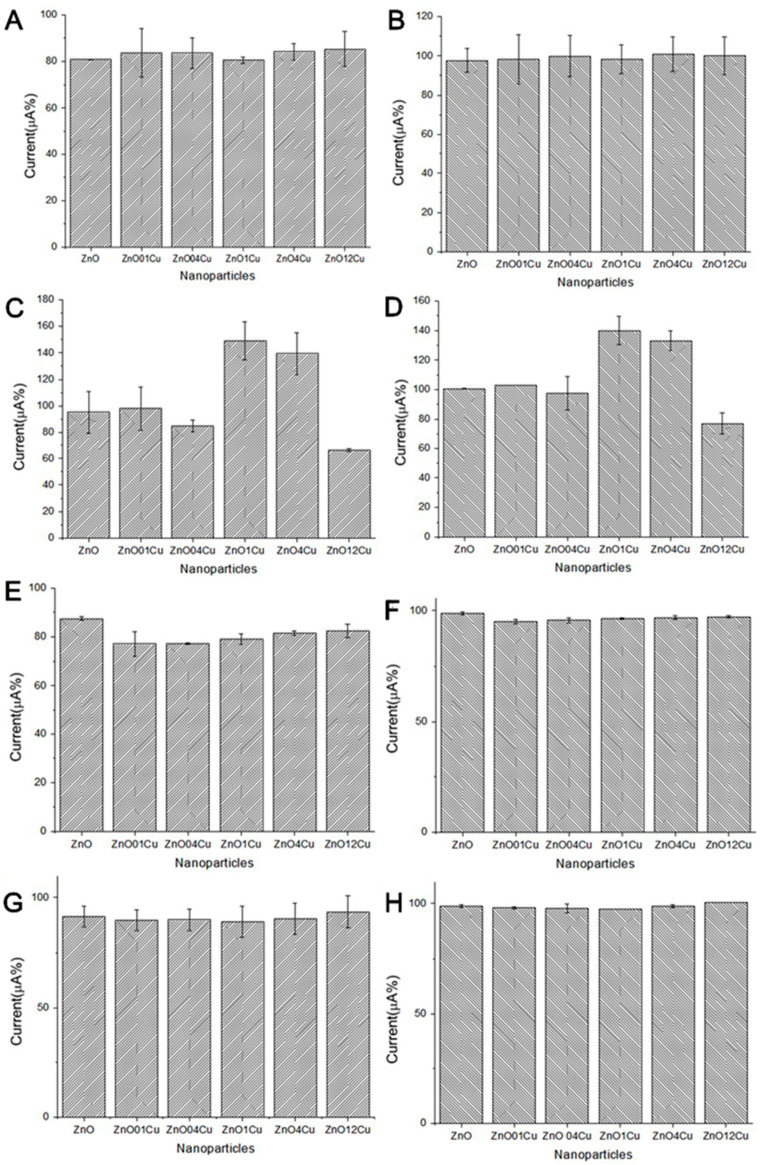
Electrochemical responses of FF/KCl in the presence of different NCs and different concentrations (mg·mL^−1^): 0.50 (**A**,**B**), 0.25 (**C**,**D**), 0.125 (**E**,**F**), and 0.625 (**G**,**H**). Oxidation peaks (first column) and reduction peaks (second column) in the cyclic voltammogram (CV) used in the construction of the column chart. Indicator electrolyte solution [Fe(CN)_6_]^−3/−4^ and KCl, electrode C110, scan rate = 100.0 mV·s^−1^.

Figure 2A,B shows the electrochemical response in the presence of different NCs and concentrations (different dilutions). The oxidation and reduction peaks were used in the construction of the column chart. The electrochemical analysis shows that, in 0.25 mg·mL^−1^, there was an increase in the current response of the redox probe (oxidation and reduction) for NC ZnO:1Cu (4th bar) and ZnO:4Cu (5th bar). It may be that these NCs provided an increase in surface area and could potentially improve sensitivity or cause minor capacitive interference in the immunosensor.

### 3.3. Immunosensor and Electrochemical Analysis

**Figure 3:**
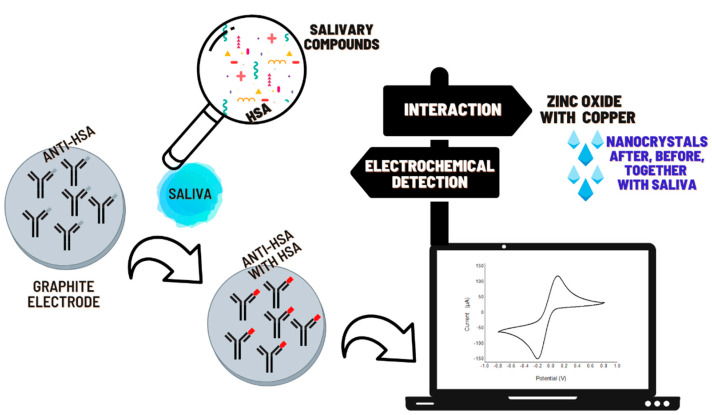
Schematic representation of the immunosensor development detection of HSA with ZnO:Cu NCs.

We investigated a modified platform with NCs to evaluate their interactions. In the sensor, we tested two different concentrations of Ab (0.744; 0.372 mg·mL^−1^), and we tested two different concentrations of casein (1% and 10%). The best results in the electrochemical sensor were found with 0.372 mg·mL^−1^ of Ab and 1% of casein. Based on this sensor construction, we made modifications to study the interaction of NCs, as shown in Figure 3.

**Figure 4:**
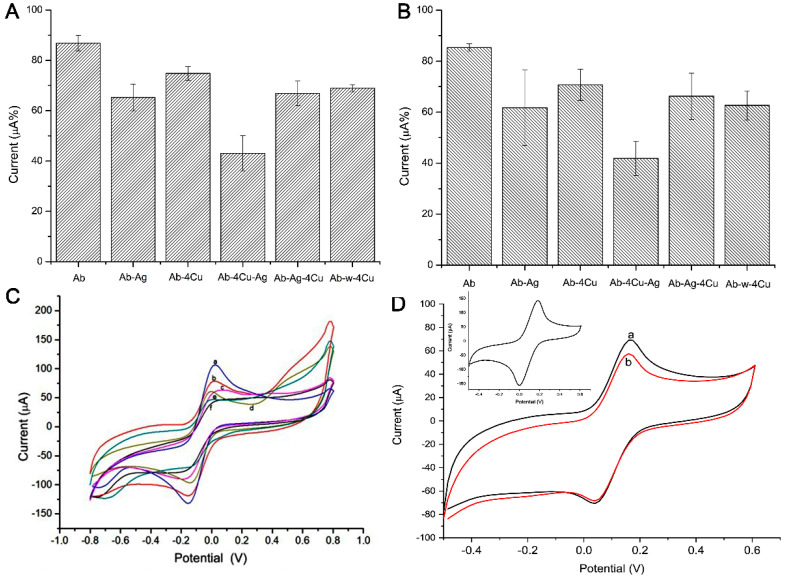
Sensor 01 and 02 presented column chart (**A**) oxidation peaks and (**B**) reduction peaks, sensor 01 (bars 1, 2, and 5) and sensor 02 (bars 3, 4, and 6), with ZnO:4Cu. Graphic (**C**) cyclic voltammetry (CV) (CV a, e, and d) and Sensor 02 (CV b, f, and c), whose peaks were used in the construction of the column chart. (**D**) CV of the sensor with saliva and ZnO:4Cu NCs mixed in one solution. The insert shows the cyclic voltammogram used to assess the electrodes’ conductivity. Indicator electrolyte solution [Fe(CN)_6_]^−3/−4^ and KCl, electrode C 110, scan rate = 100.0 mV·s^−1^. Note: all construction steps of this biosensor were shown.

Figure 4 presents the current response for each step of the biosensor construction. (A) and (B) show the oxidative and reductive analysis, in which the bar charts present the current signals. The steps were 1. immobilization of the antibody anti-HSA (Ab) (a); 2. HSA (Ag) recognition with Ab only (Ab-Ag) (b); 3. immobilization of the antibody anti-HSA (Ab) and the addition of ZnO:4Cu (Ab-4Cu) (c); 4. HSA recognition with Ab and ZnO:4Cu (Ab-4Cu-Ag) (d); 5. HSA recognition with Ab, followed by ZnO:4Cu (Ab-Ag-4Cu) (e); 6. water with Ab, followed by ZnO:4Cu (Ab-H_2_O-4Cu) (f). (C) shows the CV graphic whose peaks were used in the construction of the column chart. (D) shows the CV graphic of the sensor with saliva and ZnO:4Cu NCs mixed in one solution.

To facilitate understanding, we could consider that we had two sensors at this stage: “Sensor 01”, evaluated on bars 1, 2, and 5, and “Sensor 02”, evaluated on bars 3, 4, and 6, shown in Figure 4. Note that bars 4 and 5 present the current signals for a changing order, i.e., ZnO:4Cu addition before and after the HSA (Ag).

The most significant electrochemical current drop happened with step 4 (Ab-4Cu-Ag), compared with step 5 (Ab-Ag-4Cu), with 53.3% values, emphasizing that, here, the greater resistivity process occurred. Sensor 01 in step 2 (Ab-Ag) presented a current decrease of 77.6%. An additional test (Ab-Ag mixed with 4Cu) presented a current decrease of 78.2%, emphasizing that the specific recognition process did not occur the same way when NCs were pipetted in different orders.

**Figure 5:**
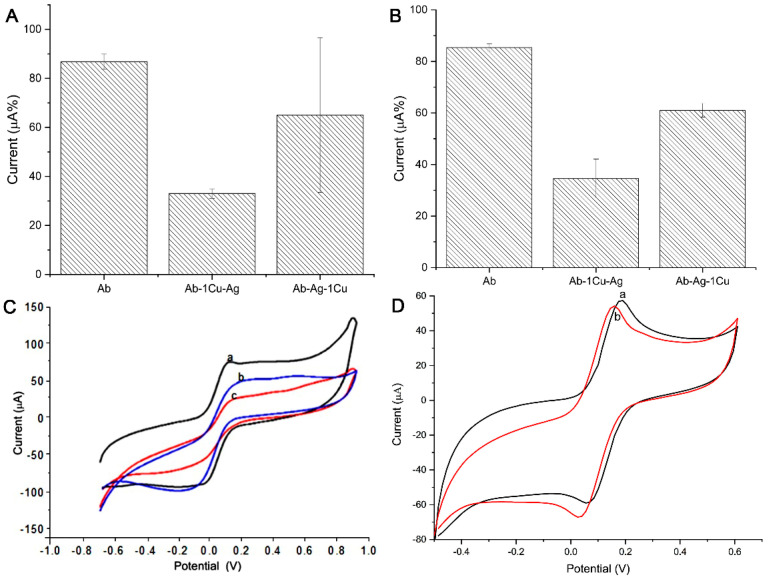
Sensor 03 presented column chart (**A**) oxidation peaks and (**B**) reduction peaks (bar 1, 2, and 3), with ZnO:1Cu. Graphic (**C**) CV (a, b, and c) whose peaks were used in the construction of the column chart. (**D**) CV of the sensor with saliva and ZnO:1Cu NCs mixed in one solution. Indicator electrolyte solution [Fe(CN)_6_]^−3/−4^ and KCl, electrode C110, scan rate = 100.0 mV·s^−1^. Note: all construction steps of this biosensor were shows.

The results presented in Figure 5A,B show the oxidative and reductive analysis made with the ZnO:1Cu, in which bar charts present the current signals. In this case, “Sensor 3”, the steps were 1. immobilization of the antibody anti-HSA (Ab) and the addition of ZnO:1Cu (Ab-1Cu) (a); 2. HSA recognition with Ab with ZnO:1Cu (Ab-1Cu-Ag) (b); 3. HSA recognition with Ab, followed by ZnO:1Cu (Ab-Ag-1Cu) (c). (C) shows the CV whose peaks were used in the construction of the column chart. (D) shows the CV of the sensor with saliva and NCs ZnO:1Cu mixed in one solution. Note that bars 2 and 3 present the current signals for a changing order, i.e., the addition of ZnO:1Cu before and after the HSA (Ag).

With Sensor 03, the most significant electrochemical current drop happened at step 2 (Ab-1Cu-Ag) compared to step 3 (Ab-Ag-1Cu), with 58.7% values, emphasizing that here the greater resistivity process occurred. An additional test (Ab-Ag mixed with 1Cu) presented a current decrease of 94.8%, emphasizing that the specific recognition process did not occur the same way when NCs were pipetted in different orders, as also noted in Figure 4.

**Figure 6:**
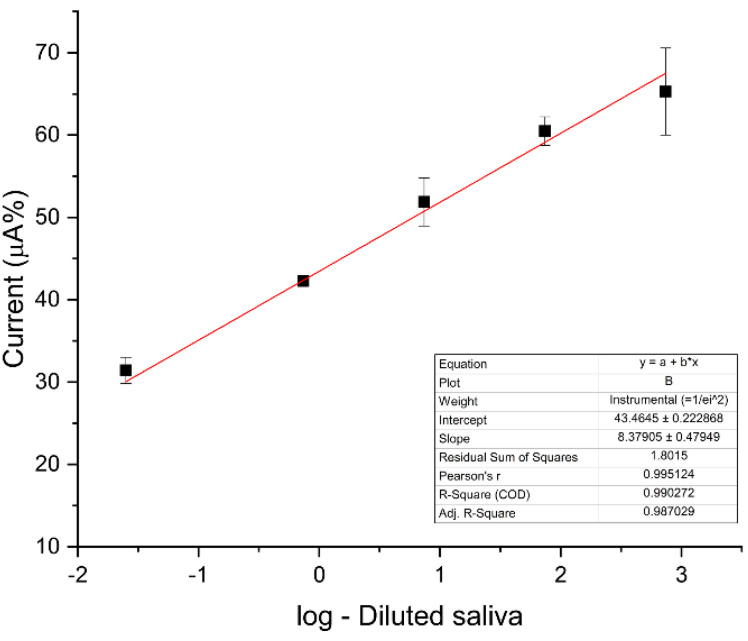
Electrochemical response for the oxidation of FF/KCl obtained after the recognition of the electrode containing the probe anti-HSA with different concentrations of HSA (log-diluted saliva). The inset shows R square, slope, and intercept values. Indicator electrolyte solution [Fe(CN)_6_]^−3/−4^ and KCl, electrode C110, scan rate = 100.0 mV·s^−1^.

Figure 6 shows the calibration curve in indirect detection evaluating the peak oxidation of FF/KCl at different saliva dilutions (pure (1 = 7.3887 U·mL^−1^, 1:10; 1:100; 1:1000; 1:10,000)). The graph shows a linear range of current peak vs. dilution. The logarithmic function (log) was used in the dilution values to achieve linearity in the graph.

Evaluating the R square, slope, and intercept, we note that the oxidation graph had better results. This plot presents the correlation coefficient of 0.990272 (for the equation: i(µA%) = 8.37905 × [saliva dilution ratio] + 43.4645), an estimated limit of detection of 0.00196 U·mL^−1^, and limit of quantification of 0.00594 U·mL^−1^. The lowest dilution values (1:30,000; 1:50,000; 1:100,000) did not show significant differences in detection (results not shown).

**Figure 7:**
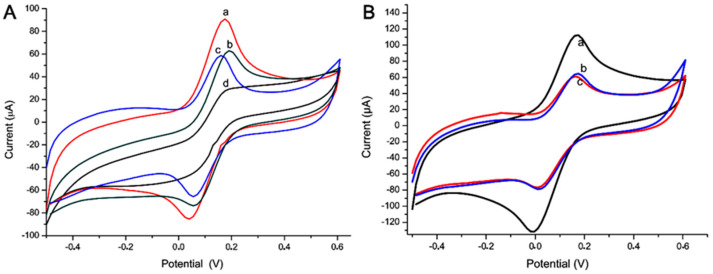
Electrochemical response of the biosensor with HSA, alpha-amylase enzyme, and trypsin for the specificity test. In this case, only saliva and AAE were detected (**A**), and (**B**) shows that TXI was not detectable. Indicator electrolyte solution [Fe(CN)_6_]^−3/−4^ and KCl, electrode C110, scan rate = 100.0 mV·s^−1^.

Figure 7 shows the current response for the specificity test with alpha-amylase enzyme (AAE) and trypsin DPCC treated, type XI (TXI); both were prepared in 0.25 mg·mL^−1^. (A) The steps were 1. immobilization of the antibody anti-HSA (Ab) (a); 2. HSA pure (Ag) recognition with Ab (Ab-Ag) (b); 3. AAE recognition with Ab (Ab-Ag) (c); 4. HSA (1:10.000) (Ag) recognition with Ab (d). (B) The steps were 1. electrode without any biomolecules (a); 2. immobilization of the antibody anti-HSA (Ab) (b); 3. TXI without recognition with Ab (c).

### 3.4. Sensor and Electrochemical Analysis

**Figure 8:**
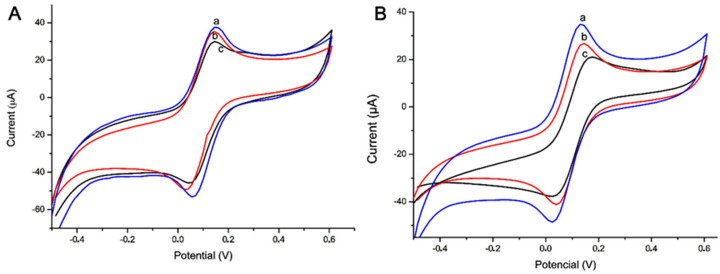
Electrochemical response of the electrode without antibody anti-HSA (Ab). In this case, it only had saliva and two types NCs in different orders. (**A**) ZnO:4Cu and (**B**) ZnO:1Cu. Indicator electrolyte solution [Fe(CN)_6_]^−3/−4^ and KCl, electrode C110, scan rate = 100.0 mV·s^−1^.

To study the interaction of NCs and saliva, a sensor without anti-HSA (Ab) was designed. Figure 8 shows the current response for each step of the other construction. (A) and (B) show the CV analysis of ZnO:4Cu and ZnO:1Cu, respectively, without steps 1 (immobilization of the antibody anti-HSA (Ab)). Step 1: adsorption of HSA (Ag) and ZnO:4Cu (Ag-4Cu) (a); step 2: adsorption of HSA (Ag) with ZnO:4Cu (Ag mixed with 4Cu) (b); step 3: adsorption of ZnO:4Cu and HSA(Ag) (4Cu-Ag) (c), and the same process was performed using ZNO:1Cu.

This analysis evaluated different responses of saliva and NCs when compared with sensors 01, 02, and 03.

## 4. Discussion

The study of the physical properties of nanomaterials is essential for the development of new technologies [43]. ZnO has a wide bandgap, chemical stability, and large exciton binding energy that outshines other oxide semiconductors for applications in diverse fields of electronics and piezoelectric outcomes [44,45]. Thus, in this work, we confirmed the formation of pure and Cu-doped ZnO nanocrystals, as well as the formation of nanocomposites (Cu-doped ZnO + CuO Ncs), and investigated the electrical responses in electrochemical sensors.

The surface roughness analysis (AFM) of the ZnO:0.4Cu NCs (Cu-doped ZnO NCs) system appeared to be much more intense when compared with ZnO NCs. Increasing the Cu^2+^ concentration to 4% (ZnO:4Cu-nanocomposite), the formation of the nanocomposite, and the synergism between the nanocrystals of Cu-doped ZnO and CuO NCs intensified the results, as shown in similar research [46,47].

We call attention to two groups of the NCs: doped (Cu-doped ZnO) and nanocomposite (Cu-doped ZnO + CuO NCs). In Cu-doped ZnO nanocrystals, copper ions were located both on the surface and inside the nanocrystal. Cu ions located on the nanocrystalline surface allowed interaction with specific biomolecules, facilitating interaction, as well as promoting mass transfer, promoting electron transfer, and avoiding photo-corrosion of nanocomposites, which enhanced its efficiency [48,49,50].

In the current study, an attempt was made to develop an immunosensor. Starting from the principle that the choice of NCs must occur from its interaction with all the molecules of a sensor, we started the electrochemical evaluations. These indicated the NCs with better electrochemical conductivities. The current responses of the two NCs, ZnO:1Cu (Cu-doped ZnO) and ZnO:4Cu (Cu-doped CuO-ZnO + CuO NCs), occurred because the first one presented maximum doping, and the second was the composite with the lowest concentration. This high conductivity occured due to copper segregation in the NC contours, causing a drop in resistance, indicating that the Cu^2+^ ions inserted in the Zn network played the role of an acceptor-type impurity [51].

In the development of a biosensor, we used the same specific polyclonal antibodies, purified by immunoaffinity, as described in others’ works [24,52]. This sensor was improved using anti-HSA diluted 100 times in dw. Our results showed better electrochemical salivary enzyme detection in this concentration, and even specificity and selectivity tests were evaluated in the recognition Ab-Ag process, shown above.

The redox pair, potassium ferrocyanide/potassium ferricyanide, acted efficiently as an indicator on the electrode surface, resulting in an efficient change of the peak current in the presence of specific NCs and biomolecules. The electrochemical analysis, the values oxidation, and reduction could be used for diagnosis analysis in cyclic voltammetry (first cycle) [53,54].

In this work, tests revealed that saliva presented a more efficient diagnosis behavior in oxidation without NCs. The presence of ZnO:4Cu or ZnO:1Cu, shown in Figure 3 and Figure 4, produced a greater drop in current when NCs were placed on the sensor before saliva.

These NCs, pipetted after Ab but before the saliva sample, altered the specificity. This result occurred because the sensor had free copper ions connectable to HSA. When the NCs were added after the saliva, we observed a current drop during the Ab-Ag recognition. We also observed that the presence of NCs did not significantly increase the current, showing that the specific coupling was partially interfered with, owing to the interaction of Cu^2+^ ions.

The third example was of that when the NCs were added together in the saliva. We did not observe a current drop during the recognition. The Cu^2+^ ion bound to HSA at the same site as the Ab binding, blocking the Ab-Ag interaction.

The electrochemical analysis presented more selectivity in the presence of NC ZnO with Cu after the saliva. The NCs collaborated to evaluate how the interference of other NCs present in saliva occurred, using small amplitude voltage.

This result could be explained because salivary proteins, including amylase, have metal-binding capacities like Cu^2+^ and Zn^2+^ [27,55,56].

The Cu^2+^ present in the surface of ZnO NCs or CuO NCs can bind with this protein through electrostatic interaction following a mechanism similar to Ca^2+^. Studies have shown that alpha-amylase has two metal-binding sites, one exclusively for Ca^2+^ and the other for Cu^2+^ and Fe^3+^ [28,56]. These reports corroborated our findings and indicated the intense interaction of Cu^2+^ with human salivary alpha-amylase.

The calibration curve had better result in the oxidation test. In this case, the redox probe could produce the tests when this reagent lost electrons during the electrochemical reaction.

The specificity of this immunosensor was validated with the enzyme TXI. Commercial alpha-amylase had the same capacity to recognize a real sample, confirming molecular recognition using an electrochemical technique [57].

To assess this interaction of alpha-amylase with Cu^2+^ ions, we developed a sensor without Ab. Thus, we were able to evaluate that the NCs, when placed before the saliva, really generated an interaction with the HSA (biggest drop in the current). However, in the absence of Ab, where the NCs were placed after the saliva, we observed an absence of interaction due to allosteric impedance of the Cu^2+^ binding sites with HSA that was directly adsorbed on the electrode. Differently, it occurred when the NC was mixed with saliva, and it freely bound to HSA. We observed an intermediary current flow, proving the interaction between them. Therefore, based on the results achieved, we can say that we have an electrostatic interaction model between HSA and Cu^2+^ that can be evaluated in this immunosensor.

## 5. Conclusions

We presented a new differential analysis of nanoparticles for better recognition of biological elements, which imparted different currents and potentials in electrochemical detection, implying better diagnostic accuracy.

Challenged by the countless works in electrochemistry using nanocrystals, we evaluated the results obtained in this work and provided opportunities for reflection based on experimental evidence.

We must evaluate the ZnO:Cu NCs, the effect of Cu concentration on physical and chemical properties, and the different ways to put them on the sensors to avoid erroneous interpretations of the results.

The results showed that the produced immunosensor showed exciting properties, such as good selectivity and sensibility. Thus, it is a promising technique of molecular analysis, and further studies of this advanced technology will extend the system to the determination of other biomolecules in saliva samples.
